# Genetic characterization of equine arteritis virus associated with outbreaks in the UK, 2019

**DOI:** 10.1099/jgv.0.002181

**Published:** 2025-12-03

**Authors:** Sushant Bhat, Siva Karunakaran, Jean-Pierre Frossard, Bhudipa Choudhury, Falko Steinbach

**Affiliations:** 1 WOAH Reference Laboratory for EVA, Virology Department, Animal and Plant Health Agency, Weybridge, KT15 3NB, UK

**Keywords:** arterivirus, equine viral arteritis, phylogenetic analysis, whole-genome sequencing

## Abstract

Equine arteritis virus (EAV) is the causative agent of equine viral arteritis, a notifiable respiratory and reproductive disease of equids that causes significant losses to the equine industry. This study presents a comprehensive analysis of two EAV outbreaks in the UK in 2019, combining virus isolation, sequencing and phylogenetic analysis to provide a holistic understanding of EAV dynamics in these outbreaks. Genetic characterization revealed that all outbreak strains were similar to viruses detected in the UK and Europe from 2004 to 2011, belonging to phylogroup D and clustering in two groups as expected based on epidemiological profiling. Bayesian phylogenetic analysis indicated the direction of transmission. The 2019 EAV strains showed maximum variability in glycoprotein (GP) 3, followed by GP2, non-structural protein 2, GP4 and GP5, with one strain displaying a unique truncation in GP4 at position 149, a feature not previously identified in arteriviruses. Polymorphisms in the CXCL16 gene have been implicated in differential susceptibility to the establishment of long-term carrier states of EAV in stallions. Genotypic analysis of the CXCL16 gene revealed that one horse possessed the homozygous genotype associated with resistance to persistent infection. In contrast, the remaining four horses exhibited the heterozygous genotype, which has been linked to an increased risk of developing a long-term carrier state and contributing to ongoing viral transmission. All infected horses exhibited the presence of neutralizing antibodies in their serum. This study underscores the importance of early detection of silent infections to reduce the spread and prevent clinical outbreaks.

## Data Availability

The full genome sequences have been submitted to GenBank, with the accession numbers listed in Table 1.

**Table 1.** Details of the EAV-positive horses showing the presence of viral RNA in semen and virus nAbs in serum

Horse no.	Sample ref.	Date of sampling	PCR*	*Ct* value	Virus isolation	WGS available (accession no.)	Serum nAb titre
1†	GB_009361_2019	11 March 2019	+	38.3	No	No	3,072
	GB_010227_2019	18 March 2019	+	35.9	No	No	na
2†	GB_009362_2019	11 March 2019	+	22.9	**Yes**	Yes (PP262573)	4,096
	GB_010228_2019	18 March 2019	+	21.2	No	Yes (PP262575)	na
3†	GB_009363_2019	11 March 2019	+	22	**Yes**	Yes (PP262574)	1,536
	GB_010229_2019	18 March 2019	+	21.8	No	No	na
4‡	GB_015276_2019	30 April 2019	+	24.7	No	Yes (PP262576)	2,048
5§	GB_024365_2019	25 July 2019	+	25.6	No	Yes (PP262577)	3,072

na – Sample not available.

*ORF5, ORF7.

†, ‡ and § represent horses from Dorset, Devon and Shropshire, respectively. Horses from Dorset were semen sampled twice with a gap of 7 days (paired analysis).

## Introduction

Equine arteritis virus (EAV) is a major equine pathogen responsible for a contagious respiratory and reproductive disease known as equine viral arteritis (EVA) [1]. EAV (formally classified as species *Alphaarterivirus equid*) belongs to the genus *Alphaarterivirus*, family *Arteriviridae* and order *Nidovirales* and is an enveloped, linear, single-stranded, positive-sense RNA virus [2]. The EAV genome comprises at least ten ORFs that encode different structural and non-structural proteins (nsp) [3]. EVA can have a significant economic impact on the equine industry due to abortions in pregnant mares and through the establishment of a carrier/persistent state in stallions [4,5]. Despite neutralizing antibodies (nAbs) in their blood, carrier stallions continuously shed virus in their semen over a variable period [6], resulting in strain diversity, with corresponding differences in their genotype and phenotype [7,8]. EVA usually manifests as a subclinical disease; however, clinical signs, including severe respiratory illness, fever and reproductive complications, may be seen in cases, which vary depending on the genotype of the virus, dose of the virus and route of infection [9]. EAV infection evokes virus-specific antibodies; thus, pre-breeding screening of stallions for the presence of EAV antibodies is recommended by the UK Horserace Betting Levy Board [10].

EVA was first identified in the mid-20th century and has since been recognized as a disease of global concern, with sporadic outbreaks occurring globally [11] except for Iceland, Japan and New Zealand, which have declared themselves free from EAV. During the last decade, EAV has continued to be reported, for example, in Algeria [12], Germany [13], Serbia [14] and South America [15], with some reports of seropositive horses in Costa Rica, France, Serbia and Spain [16,19]. The UK experienced its first incursion of EAV in 1993 from a non-thoroughbred stud from Poland [20]. The disease was made notifiable in the UK in 1995, and the detection of EAV in a horse is classed as an outbreak [21]. Subsequently, there were reports of EAV-seropositive equines [22], and EAV infections were identified in 2004 and 2010 in stallions imported from the Netherlands [23,24]. Prior to the 2019 outbreak, the last recorded case in the UK was a genotypically divergent EAV identified in an imported Spanish stallion in 2012 [25].

In early 2019, following routine pre-breeding serological testing, four apparently healthy stallions from the southern counties of Devon and Dorset tested positive for EAV [26]. Epidemiological investigations indicated no spread to the wider UK equine population [27], although another horse in the northern county of Shropshire also tested positive in July 2019 [28]. In this study, virological characterization of all 2019 outbreak samples was undertaken.

## Methods

### Clinical samples

Semen and blood samples from apparently healthy stallions in Devon, Dorset and Shropshire were submitted to the Animal and Plant Health Agency, UK. Semen samples were centrifuged at 117 ***g*** at room temperature for 5 min to collect seminal plasma. Blood samples were left overnight for the separation of serum. Sera were harvested and stored at −80 °C until further use.

### Nucleic acid extraction

RNA was extracted from the seminal plasma using TRIzol reagent (Thermo Fisher Scientific) and QIAamp Viral RNA Mini kit (Qiagen) using protocols provided by the manufacturers. The samples were first treated with TRIzol, following which the aqueous phase was removed and then processed using the QIAamp Viral RNA Mini kit.

### ORF5 RT-PCR and ORF7 qRT-PCR

Reverse transcription PCR (RT-PCR) targeting the ORF5 gene and quantitative reverse transcription PCR (qRT-PCR) targeting the ORF7 gene of equine arteritis virus (EAV) were carried out using gene-specific primers, using previously described methods [29,30]. *ß* actin was used as an internal control for RNA extraction [31].

### ORF5 sequencing

The ORF5 sequencing was carried out by the Sanger dideoxy chain termination method as described previously [25]. To determine the homology between the ORF5 sequences, the pairwise distance between ORF5 sequences was calculated using the maximum composite likelihood in MEGA X [32]. Percentage nucleotide identity (PNI) was calculated using the formula PNI (%)=(1−pairwise distance)×100. The data were plotted, and a heatmap matrix was generated using the webserver ‘matrix2png’ [33].

### Next-generation sequencing

Total RNA was extracted from semen samples, as described above. The RNA was treated with TURBO^™^ DNAse (Invitrogen), and the library was prepared using Nextera XT DNA Library Prep kit (Illumina) and sequenced using the MiSeq System (Illumina). EAV reference sequence (RefSeq) Bucyrus (GenBank accession no. NC_002532.2) was used as a reference genome for mapping paired-end Illumina reads using Geneious mapper in Geneious Prime 2023.2 (https://www.geneious.com/). The consensus full-length genomes were identified by setting a threshold of 70%.

### Phylogenetic analysis

**Fig. 1.**
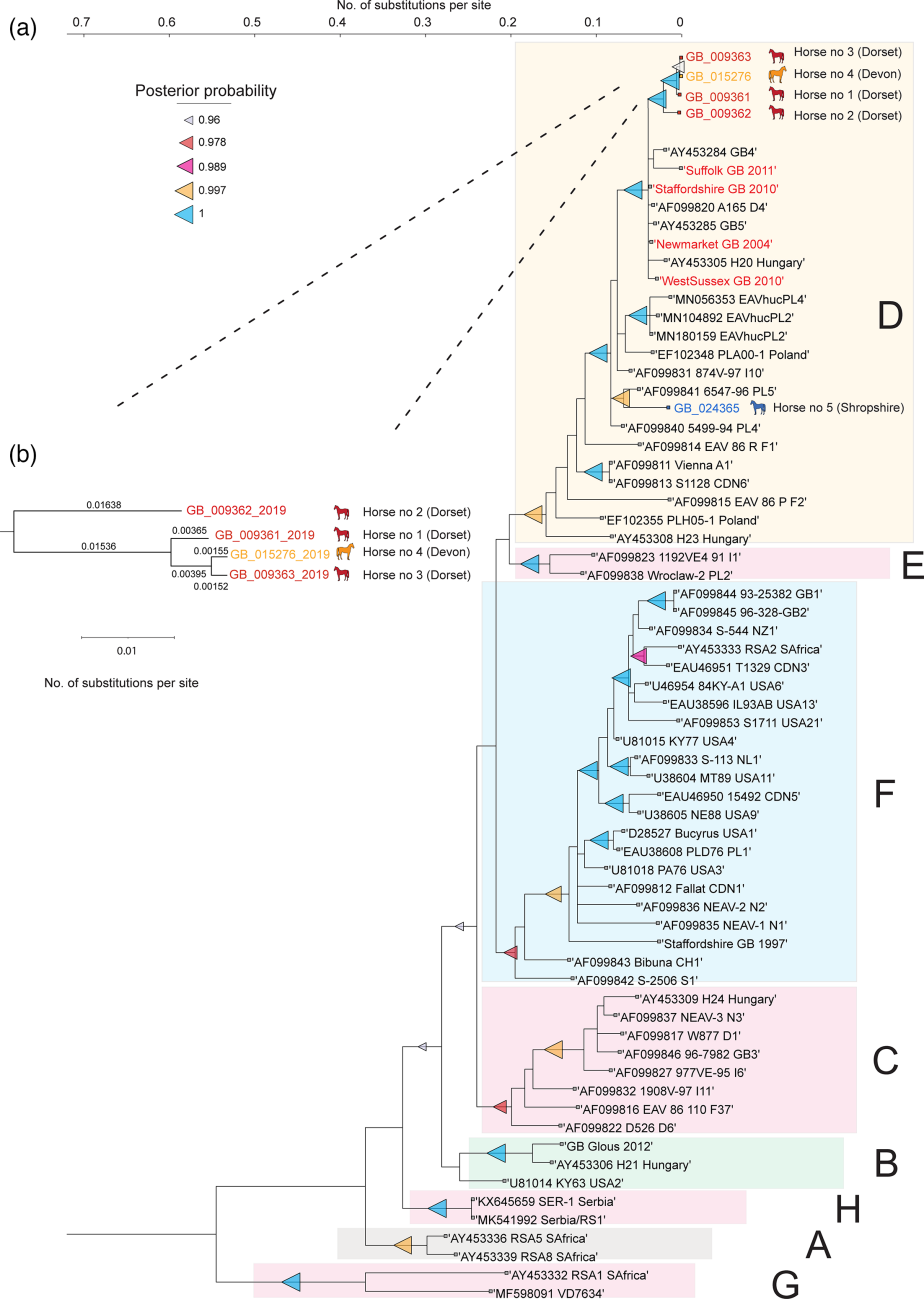
Bayesian phylogenetic analysis of ORF5 sequences of the EAV strains identified in 2019 in the UK using MCMC method (a). The ORF5 sequences of 2019 EAV strains were aligned using clustalw with the reference sequences downloaded from GenBank. The molecular clock test was performed using MEGA XI by comparing the ML value for the given topology with and without the molecular clock constraints under the General Time Reversible model (+G+I). The Bayesian estimation of phylogeny was done by MrBayes using a ‘relaxed unconstrained branch length’ as a prior. The donkey isolate (MF598091) was kept as an outgroup. The final Bayesian tree was visualized, with branch support indicated by posterior probabilities. The nodes showing the posterior probability of 1 are marked and assigned to groups A–H. This analysis involved 67 ORF5 nt sequences. The number of substitutions per site for each of the 2019 EAV sequences is indicated on the subtree (**b**). The tree was rooted to the midpoint.

Phylogenetic analysis was carried out using ORF5 and whole-genome nucleotide sequences. For ORF5 phylogenetic analysis, partial ORF5 sequences belonging to different phylogroups [25], previously reported from other EAV outbreaks around the world (and listed in Fig. 1), were aligned using clustalw in MEGA X [32]. For whole-genome phylogenetic analysis, the genome sequences of 2019 EAV strains were aligned with the full genome sequences available in GenBank using clustalw. The Bayesian estimation of phylogeny was carried out using MrBayes [34] plugin in the Geneious Prime version 2025.0.3. The GTR substitution model with gamma rate variation was selected. Markov Chain Monte Carlo (MCMC) run was performed for 1,100,000 generations, sampling every 200 generations. The first 100,000 trees were discarded as burn-in, and the remaining trees were used to generate a 50% consensus tree. The analysis was performed using a relaxed molecular model by selecting the unconstrained branch length. The consensus tree was exported for downstream visualization and annotated in iTOL V7 [35]. The final Bayesian tree was visualized, with branch support indicated by posterior probabilities [36,37].

### Virus isolation

Virus isolation in Rabbit Kidney 13 (RK13) cells was attempted on qRT-PCR-positive semen samples following a WOAH-recommended protocol described in the Terrestrial Manual 2018 [1]. The presence of the virus was confirmed by virus titration and immunoperoxidase staining of the infected cells. Briefly, RK13 cells were infected with 10-fold serial dilutions of cell supernatant showing positive cytopathic effects (CPE) and incubated for 1 h at 37 °C before being washed twice with Eagle's Minimal Essential Medium (EMEM) and incubated at 37 °C (5% CO_2_) for 24 h. Cells were fixed in a PBS solution containing 20% acetone for 10 min. The cells were washed twice with wash buffer (PBS; 0.5% Tween) followed by the addition of EAV-specific antiserum (1:100) diluted in PBS; 1% Tween; 2.1% sodium chloride; serum diluent and incubated at 37 °C for 1 h. Cells were washed thrice with wash buffer, followed by the addition of Rabbit Anti-Horse IgG (whole molecule)–HRP (Sigma-Aldrich) (1:200 dilution in serum diluent) and incubated at 37 °C for 1 h. The EAV-infected cells were detected using ethyl carbazole (0.089% glacial acetic acid, 0.289% sodium acetate).

### Titration of the Bucyrus strain

The prototype EAV Bucyrus strain was 10-fold serially diluted in EMEM (containing 1 mM sodium pyruvate, 20 mM HEPES and 1X Penicillin Streptomycin), and 100 µl of each virus dilution was added to the wells of the microplate containing RK13 cells; each virus dilution was added to a separate column containing eight wells. Two columns were kept as non-infected cell controls. The plate was incubated at 37 °C for 72 h. To calculate the virus dose for the serum neutralization test (SNT), wells exhibiting CPE over 25% in any field were identified under a microscope and marked as positive. The virus titre (TCID_50_) was determined by the Reed and Muench method [38].

### Neutralization test

SNTs were carried out to determine the presence of nAbs in serum. For the SNT, the serum samples, including known EAV-positive antiserum and EAV-negative antiserum, were heat treated at 56 °C for 30 min to inactivate the complement. Twofold serial dilutions of the antisera were made (from 1/32 to 1/65,536) in EMEM (containing 1 mM sodium pyruvate, 20 mM HEPES and 1X Penicillin Streptomycin). Twenty-five µl per well of each serum sample was added to two rows of a microtitre plate, and each sample was tested in duplicate. Fifty µl of RK13 cell suspension containing 3×10^5^ cells in EMEM (with 20% FBS, HEPES and 1 X Penicillin Streptomycin) was added to each well containing antiserum. Twenty-five µl of EAV Bucyrus strain (200TCID_50_) was added to each well, and plates were incubated at 37 °C for 72 h with 5% CO_2_. The wells showing CPE were identified by observing the plates under an inverted microscope. Positive wells were marked when CPE was present in more than 25% of the field. The reciprocal of the highest dilution of antiserum, which could prevent virus-induced CPE, was taken as a serum nAb titre.

### Glycosylation prediction and analysis of neutralization sites

The potential N-linked glycosylation sites were identified using the NetNGlyc web server [39] to compare glycosylation sites in glycoprotein (GP) 3 and GP5. The Asn in the Asn-Xaa-Ser/Thr sequons (Asn – Asparagine, Xaa – Any amino acid, Ser – Serine, Thr – Threonine) predicted to be N-glycosylated were identified using a 0.5 cut-off. The neutralization sites were analysed for conserved amino acids and represented using Weblogo [40].

### Selection analysis

Selection pressure analysis was done on GP3 and GP5 proteins using single-likelihood ancestor counting (SLAC) [41], fixed effects likelihood (FEL) [41], mixed effects model of evolution (MEME) [42] and fast unconstrained Bayesian approximation (FUBAR) [43] methods in the datamonkey web application (https://www.datamonkey.org). For SLAC analysis, non-synonymous (dN) and synonymous (dS) substitution rates on a per-site basis were identified using a combination of maximum likelihood (ML) and counting approaches. Sites with a dN/dS >1 were considered to undergo positive selection, using a posterior probability value threshold of 0.1. For FEL analysis, codons where the relative non-synonymous substitution rate for tested branches (*β*) exceeded the relative non-synonymous substitution rate (*α*), showing *β*>*α* at a *P* value <0.1, were considered to undergo positive selection. For MEME analysis, sites where the non-synonymous rate under positive selection (*β*+) exceeded *α* with a *P* value <0.1 were considered to be under positive selection. For FUBAR analysis, codons where the mean posterior non-synonymous substitution rate (*β*) exceeds the mean posterior synonymous substitution rate (*α*) were identified. A high Bayes factor was used as evidence for positive selection at a given site. The sites which showed evidence of positive selection using all four methods were considered to undergo positive or diversifying selection.

### Analysis of the CXCL16 gene variants in stallions

Total DNA was extracted from semen samples collected from each stallion using QIAamp DNA Mini kit (Qiagen) as per the manufacturer’s instructions. PCR reactions were performed using PfuII Ultra Hotstart Master Mix (Agilent). Each reaction mix consisted of 0.4 µM of each primer (CXCL16-F and CXCL16-R11) [44], 1×master mix and 2 µl of DNA in a total volume of 50 µl. The 280 bp CXCL16 amplicons were gel extracted and sequenced using the Sanger dideoxy chain termination method. The sequenced amplicons were aligned to the reference equine CXCL16 sequence (GenBank ref: XM_001504756.6) and genotyped based on the presence of specific amino acids at positions 40, 49, 50 and 52. The presence of homozygous alleles (Y, D, F and E) at specified positions was marked as resistant genotype ‘EqCXCL16R’, while the presence of heterozygous alleles (Y/F, D/H, F/I, E/K) associated with the long-term carrier status was designated ‘EqCXCL16S’. All analyses were performed using Snapgene.

## Results

### Disease identification: qRT-PCR and virus isolation

The infections were detected as a consequence of pre-breeding testing in clinically healthy non-thoroughbred stallions. Semen and blood samples were tested for the presence of viral RNA by qRT-PCR and antibodies by SNT, respectively (Table 1). Paired semen samples from the infected horses tested positive using the qRT-PCR targeting ORF7. Samples from Dorset Horse 1 were weakly positive, with *Ct* values ranging from 35.9 to 38.3; the remaining six samples (from Dorset Horses 2 and 3, Devon Horse 4 and Shropshire Horse 5) were strongly positive, showing *Ct* values in the range of 21.2–25.6 (Table 1). All samples were then tested by virus isolation, but the virus could only be isolated from two semen samples belonging to Dorset Horses 2 and 3.

### Phylogenetic analysis of the viruses

Initial phylogenetic analysis was carried out using ORF5 nucleotide sequences derived from the EAV outbreak sequences (1 per animal) and 62 reference genomes belonging to phylogroups A-G [25]. Bayesian phylogenetic analysis carried out by the MCMC method showed that four out of five 2019 outbreak strains clustered together, showing a posterior probability of 1, while the GB_024365_2019 strain sampled from Shropshire Horse 5 was genetically distinct (Figs 1 and S1, available in the online Supplementary Material). All 2019 outbreak strains belong to phylogroup D [25] forming a monophyletic group of sequences identified in the UK between 2004 and 2011 and clustered together with Polish and Hungarian isolates. It should be noted that this analysis resulted in a new genotype H that arose from the additional EAV isolates since 2015 from Serbia [25]. Posterior probability confirmed the reliability of trees, which was also corroborated by ML analysis (Fig. S2). Reference sequences clustered into different phylogroups with posterior probability ranging from 0.96 to 1 and bootstrap values ranging from 60 to 100% (Fig. S3).

**Fig. 2.**
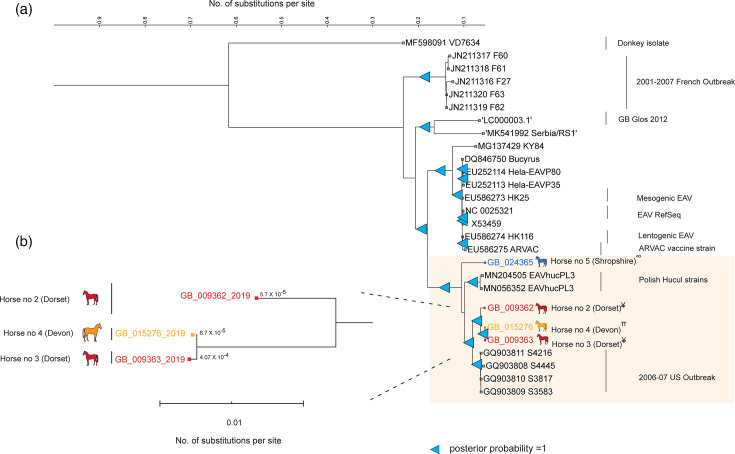
Bayesian whole-genome phylogenetic analysis of EAV strains identified in 2019 in the UK using MCMC method (a). The genome sequences of 2019 EAV strains were aligned with the full genome sequences available in GenBank using clustalw. The molecular clock test was performed using MEGA XI by comparing the ML value for the given topology with and without the molecular clock constraints under the General Time Reversible model (+G+I). The Bayesian estimation of phylogeny was done by MrBayes using a ‘relaxed unconstrained branch length’ as a prior. The donkey isolate (MF598091) was kept as an outgroup. The final Bayesian tree was visualized, with branch support indicated by posterior probabilities. This analysis involved 28 full genome nucleotide sequences. There were a total of 12,454 positions in the final dataset. The number of substitutions per site for each of the 2019 EAV sequences is indicated on the subtree (**b**). The coloured solid spheres indicate the 2019 UK EAV strains, with red indicating the EAV sequence identified in horses from Dorset, orange indicating the EAV sequence from the Devon stallion and blue indicating the EAV sequence identified in the stallion from Shropshire. The tree was rooted to the midpoint.

Bayesian phylogenetic analysis was also undertaken with whole-genome sequences of strains identified in 2019 and 36 complete genomes which were representative of all the EAV sequences available on GenBank. This complete genome-based phylogenetic analysis confirmed that the Shropshire strain was phylogenetically distinct (outgroup) compared to other 2019 UK strains (Figs 2 and S4). Here, the 2019 UK outbreak strains clustered with the EAVs identified in the USA in the 2006–2007 outbreak and with Polish EAV strains identified in Hucul horses [45]. Reference genomes from donkeys appeared to be the most distant in all analyses.

Bayesian estimation combining whole-genome phylogeny and number of nucleotide substitutions per site indicated that within the Dorset/Devon cluster, Horse 2 was first infected and transmitted the virus to Horses 1, 3 and 4 (Figs1b  2b). The precise order of transmission cannot be resolved through this analysis, but it seems likely that transmission occurred on at least two separate occasions, with Dorset Horse 1 being an intermediate.

### Comparison of genome sequences

Whole-genome sequences could be recovered from four of the five horses. The comparison showed that Dorset strains (GB_009362_2019 and GB_009363_2019) had a genome length of 12,704, while the Shropshire strain (GB_024365_2019) was 12,700nt in length. The Devon strain (GB_015276_2019) showed an incomplete EAV leader sequence with a 12,697 nt genome length. The sequences were compared with the GB_Glos_2012 genome, which was 12,702 nt in length.

**Fig. 3.**
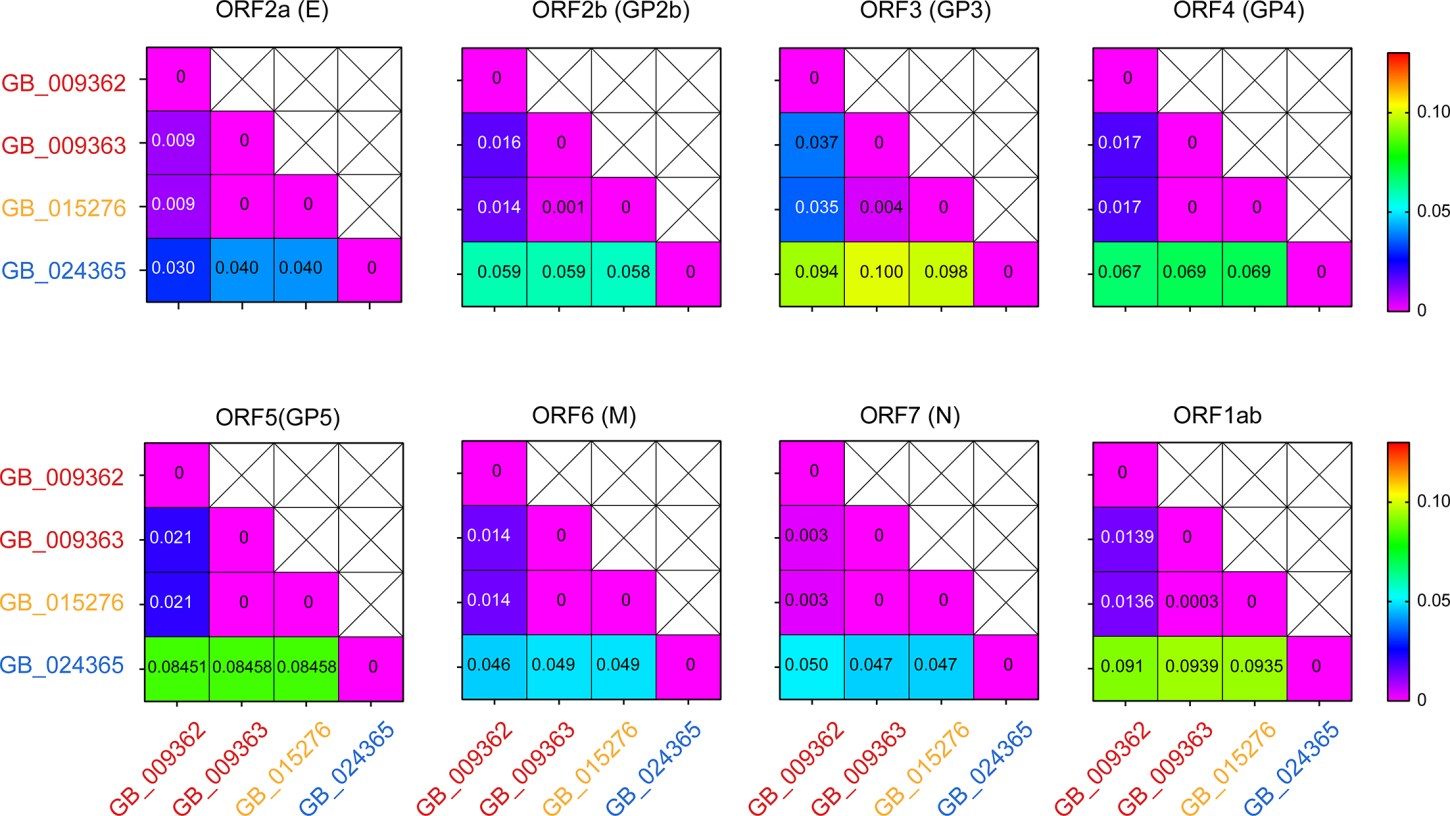
Estimates of evolutionary divergence between different ORF sequences of the equine arteritis strains identified in 2019 in the UK. The number of nucleotide substitutions per site from different ORF sequences is shown. Pairwise distance(s) were obtained by a bootstrap procedure (1,000 replicates) in MEGA X, and the values are shown below the diagonal in each cell. The pairwise distance was plotted as a heatmap using GraphPad Prism 8. The names of the strains used in the analysis are shown on the left side and the bottom of the heat map. E: envelope protein; M: membrane protein; N: nucleocapsid protein.

Among the 2019 strains, the Shropshire strain showed less similarity to the Devon and Dorset strains, with the most variability found in ORF3 (GP3) and ORF1ab, followed by ORF5 (GP5), ORF4 (GP4), ORF2b (GP2b), ORF7 (N), ORF6 (M) and ORF2a (E) (Fig. 3). Within ORF1ab, maximum variability was seen in nsp11, nsp2 and nsp12, followed by nsp4, nsp5, nsp6, nsp3, nsp7, nsp9, nsp10, nsp8 and nsp1 (Fig. S5). The per cent similarity between the Dorset strain (GB_015276_2019) and the two Devon strains (GB_009362_2019 and GB_009363_2019) was 99.72 and 99.89%, respectively, with maximum divergence in GP3 followed by GP5. The GB_009362_2019 and GB_010228_2019 genomes (identified in paired semen samples from Horse 2) differed from one another due to nine synonymous mutations (data not shown), and importantly a non-synonymous mutation (P194S) was observed in the consensus sequence of nsp2. The whole-genome sequences of 2019 EAV strains did not show any indels in the ORF1ab polypeptide, unlike the one amino acid deletion observed in nsp2 of GB_Glos_2012 [25]. Phylogenetic analysis confirms that the EAV from Horse 2 has an ancestral linkage to the viruses in Horses 3 and 4.

The GP4 mature peptide of the Shropshire strain showed a three amino acid truncation due to a premature stop-codon incorporated due to C/T transition in the codon at position 149, while the strains from Devon and Dorset had full-length GP4. The C/T transition at position 149 was observed in more than 70% of sequencing reads.

A percentage homology comparison between different proteins of the 2019 EAV strains with reference to the GB_Glos_2012 genome revealed nsp6 protein was most conserved with 100% homology, followed by the nucleoprotein (N), while the GP3 protein showed the highest variability (Fig. S6).

### Glycosylation and selection analysis in GP5 and GP3

**Fig. 4.**
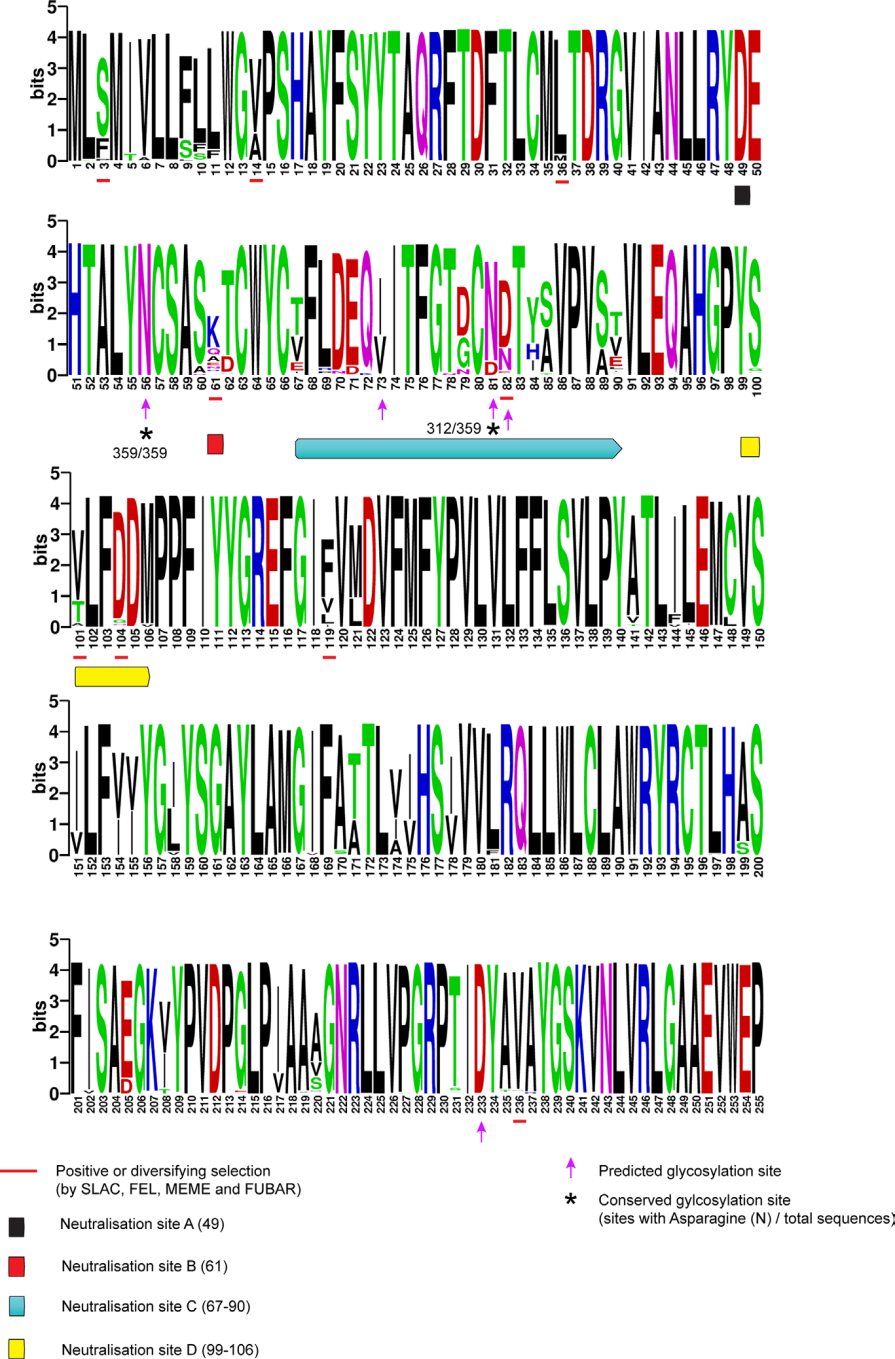
Amino acid analysis of GP5 showing neutralization sites A, B, C and D, glycosylation and selection analysis. Each sequence logo consists of a letter or a stack of letters representing an amino acid at a particular position. The overall height of the stack (shown as bits) indicates the sequence conservation at that position. The height of the symbols within each stack represents the relative frequency of each amino acid at that position. The numbers below the sequencing logo indicate amino acid position. Amino acids are coloured according to their chemical properties: polar amino acids (**G, S, T, Y, C, Q, **N) are green, basic (**K, R, **H) are blue, acidic (**D, **E) are red and hydrophobic (**A, V, L, I, P, W, F, **M) amino acids are shown as black. Neutralization sites A, B, C and D are indicated with coloured bars. Glycosylation sites are indicated with an arrow. Conserved glycosylation sites are marked with an asterisk. Sites undergoing positive selection as identified by SLAC, FEL, MEME and FUBAR are underlined with red.

All available EAV ORF5 (encoding GP5) and ORF3 (encoding GP3) sequences were retrieved from GenBank and aligned using muscle. Sequences having ambiguous bases were removed. A total of 359 GP5 and 300 GP3 sequences were subsequently analysed to identify potential N-linked glycosylation sites and to assess selection pressure. For GP5, the glycosylation site identified at position 56 was conserved in all sequences (Fig. 4). A second site at position 81 was relatively conserved, present in 312 sequences. Other potential sites at positions 73, 82, 233 and 240 were non-conserved and observed in only a few sequences. Notably, the GP5 of 2019 EAVs had only two glycosylation sites, namely at amino acid positions 56 and 81.

Sequence alignment of GP5 ORFs showed that 2019 EAV outbreak strains differed among themselves (Fig. S7A) and previous UK EAV outbreak viruses (Fig. S7B) at neutralization sites C and D. The sites C and D harbour the major neutralizing epitopes of EAV by forming a conformational dependent neutralization domain as proposed in some studies [46,47]. Selection pressure analysis of full-length GP5 using SLAC, FEL, MEME and FUBAR (Table S1) showed that site 61 in neutralization site B, site 82 in neutralization site C and sites 101 and 104 in neutralization site D showed positive or diversifying selection. This suggests that the neutralization (but not glycosylation) sites in GP5 are under evolutionary pressure from the host immune response, leading to mutations that help the virus persist or spread.

**Fig. 5.**
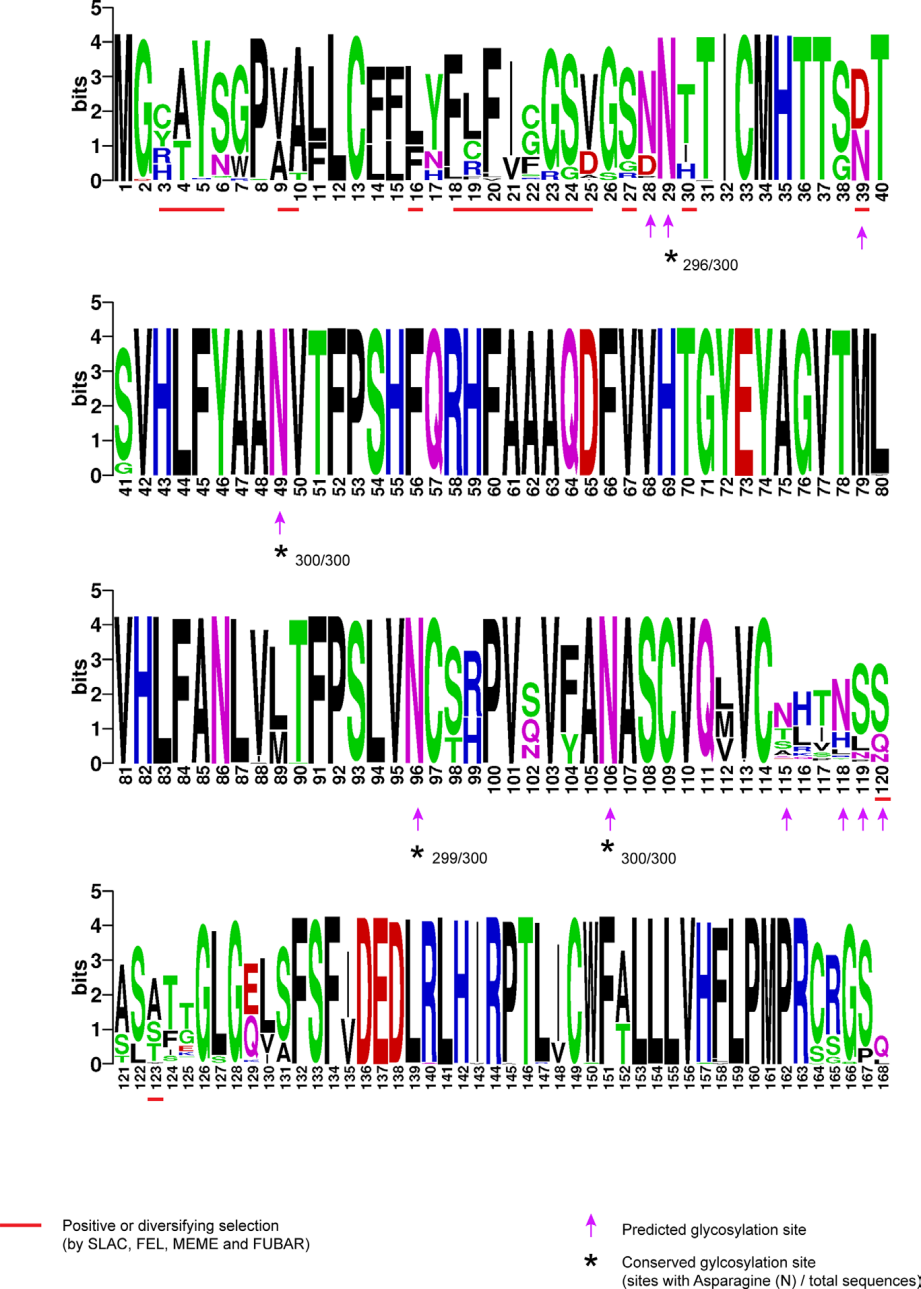
Amino acid analysis of GP3 showing glycosylation and selection analysis. Each sequence logo consists of a letter or a stack of letters representing an amino acid at a particular position. The overall height of the stack (shown as bits) indicates the sequence conservation at that position. The height of the symbols within each stack represents the relative frequency of each amino acid at that position. The numbers below the sequencing logo indicate amino acid position. Amino acids are coloured according to their chemical properties: polar amino acids (**G, S, T, Y, C, Q, **N) are green, basic (**K, R, **H) are blue, acidic (**D, **E) are red and hydrophobic (**A, V, L, I, P, W, F, **M) amino acids are shown as black. Neutralization sites A, B, C and D are indicated with coloured bars. Glycosylation sites are indicated with an arrow. Conserved glycosylation sites are marked with an asterisk. Sites undergoing positive selection as identified by SLAC, FEL, MEME and FUBAR are underlined with red.

For GP3, glycosylation was identified at positions 28, 29, 39, 49, 96, 106, 115, 118, 119 and 120 (Fig. 5). Among these sites, glycosylation at positions 29 (296/300), 49 (300/300), 96 (299/300) and 106 (300/300) was conserved or relatively conserved across the dataset. Positive selection was identified at least at sites 3–6, 9–10, 16, 18–25, 27, 120 and 123 (Table S2). Compared to GP5 sequences, GP3 sequences exhibited a higher proportion of glycosylation sites (5.95% vs. 2.35%) and a significantly greater number of codons under positive selection (11.9% vs. 3.52%) which implies that EAV GP3 is subject to a stronger evolutionary pressure and is evolving more rapidly than GP5.

### Antibody response

All horses showed the presence of nAbs when tested in SNT with titres ranging from 1,536 to 3,072 (Table 1).

### EqCXCL16 genotyping of EAV-infected stallions

Among the horses sampled in Dorset, Horse 1 was found to be homozygous for the EqCXCL16R allele. This allele is associated with resistance of CD3+T lymphocytes to EAV infection, thereby reducing the likelihood of the horse developing a carrier status. In contrast, all other horses were heterozygous for the EqCXCL16 allele (EqCXCL16S/EqCXCL16R), which has been linked to a susceptible phenotype [44]. These horses were therefore at an increased risk of establishing a carrier stage following EAV infection (Table 2).

**Table 2.** Details of CXCL16 genotyping of infected horses for identification of EAV carrier stage

Horse no.	Sample ref.	CXCL16 amino acid at position				CXCL16 alleles	
		40	49	50	52		
1	GB_009361_2019	Y	D	F	E	CXCL16^R^/CXCL16^R^	HR
2	GB_009362_2019	Y/F	D/H	F/I	E/K	CXCL16^S^/CXCL16^R^	HS
3	GB_010229_2019	Y/F	D/H	F/I	E/K	CXCL16^S^/CXCL16^R^	HS
4	GB_015276_2019	Y/F	D/H	F/I	E/K	CXCL16^S^/CXCL16^R^	HS
5	GB_024365_2019	Y/F	D/H	F/I	E/K	CXCL16^S^/CXCL16^R^	HS

The presence of homozygous alleles (Y, D, F and E) at amino acid positions 40, 49, 50 and 52 corresponds to the EAV carrier-resistant stage (CXCL16R), while the presence of heterozygous alleles (Y/F, D/H, F/I, E/K) corresponds to the EAV carrier-susceptible stage (CXCL16S/CXCL16R).

HR denotes homozygous resistant; HS denotes heterozygous susceptible.

## Discussion

EVA is a notifiable disease in equines, potentially causing significant economic loss [1,9]. In 2019, EAV outbreaks in the UK, specifically in Devon, Dorset and Shropshire, were identified through pre-breeding tests, illustrating the importance of proactive surveillance systems in detecting and managing infectious diseases.

Analysing EAV’s genetic diversity is crucial for understanding the molecular epidemiology of outbreaks and limiting disease transmission within equine populations. ORF5 of EAV is commonly used for phylogenetic analysis [29,48, 49], although GP3 [7,50] and ORF1b have also been used [51,53]. Phylogenetic analysis of ORF5 showed that the 2019 outbreak strains from Devon and Dorset were from the same epidemiological site and belonged to phylogroup D that also contains EAV strains identified in the UK between 2004 and 2011 [25]. Continued efforts to detect EAV resulted in another case identified in Shropshire in July 2019. Although the strain identified from Shropshire also belongs to phylogroup D, it formed a sub-clade with the Polish ‘PL5’ strain [29], which is phylogenetically distinct from the Devon and Dorset strains, as supported by very high posterior probability and bootstrap values. There was no history of mating or transport of the Shropshire horse to events within or outside the UK, demonstrating a missing link between the source of infection and identification of carrier status. This emphasizes the need for vigilance to better understand the dynamics of EAV transmission.

As more variability was noted in GP3 and ORF1ab (particularly in nsp5 and nsp9), compared to GP5 (Figs 3 and S5), GP5-based phylogeny may overestimate the reliability of tracing the order of infection. A comparison of evolutionary divergence among ORF3, ORF4 and ORF5 sequences of all available EAV strains also showed GP3 with more variability than GP5 and GP4 (Fig. S8). Whole-genome-based phylogenetic analysis is so far less common [54] for EAV. Moreover, commercial diagnostic testing does not mandate sequencing, and both cost considerations and low viral loads in some semen samples often lead to a preference for partial PCR-based sequencing. As a result, the number of complete EAV genomes available in the GenBank database remains limited.

Both the ORF5-based analysis and the whole-genome phylogenetic analysis showed fewer nucleotide substitutions in sequences from Dorset Horse 2 compared to Devon Horse 4 and Dorset Horse 3. Hence, in both analyses, Dorset Horse 2 was more likely to be the index horse, which was infected from an unconfirmed source, followed by the spread of infection to Devon Horse 4 and Dorset Horse 3. The role of horse 1 from Devon in the infection chain remains more uncertain due to the absence of whole genomic data, and there is no evidence supporting this horse to be the index case as assumed by the clinical epidemiological investigation. The absence of whole-genome sequences from recent EAV outbreaks makes it challenging to map the exact source of the outbreak.

The amino acid analysis of different proteins showed a unique truncation in GP4 of the Shropshire strain, which could have implications for virus assembly and host-cell attachment [55]. While deletions at the 3′ end of ORF3 are common in arteriviruses [56] and affect the 5′ end of ORF4 (GP4) due to overlapping reading frames, truncations at the carboxy-terminal of GP4 leading to a shorter GP had not been identified before. Whether the GP4 truncation has any phenotypic implication on EAV needs to be analysed further. GP3 encoded by ORF3 was found to be the most variable, as seen in other arteriviruses, like Porcine Reproductive and Respiratory Syndrome Virus (PRRSV) [57], and has been reported to encounter strong selection pressure during persistent EAV infection in a thoroughbred carrier stallion [58]. This was also corroborated by our GP3 selection analysis, which showed an increased number of sites undergoing positive selection compared to the GP5.

Glycosylation of viral proteins can have a pronounced effect on antigenicity, affect virus virulence and antibody-based neutralization [59,60]. While no change in glycosylation sites was identified in GP5 of 2019 outbreak strains compared to the previous EAV strains identified in the UK, the 2019 EAV outbreak strains differed amongst themselves at the previously proposed antibody neutralization sites, which had been identified as mutational ‘hot spots’ responsible for the emergence of phenotypic variants with altered neutralization potential [58,61]. EAV infection triggers a humoral antibody response [62]. Currently, there is only one serotype associated with GP5 of EAV, and selection analysis indicates that four sites (61, 82, 101 and 104) in neutralization sites B, C and D are undergoing positive selection. Changes in the neutralization sites reflect adaptive changes that allow the virus to evade antibody recognition. The positively selected sites in neutralization sites B, C and D may therefore represent an immune-driven hotspot of variation that warrants continued monitoring as more EAV outbreaks emerge.

**Fig. 6.**
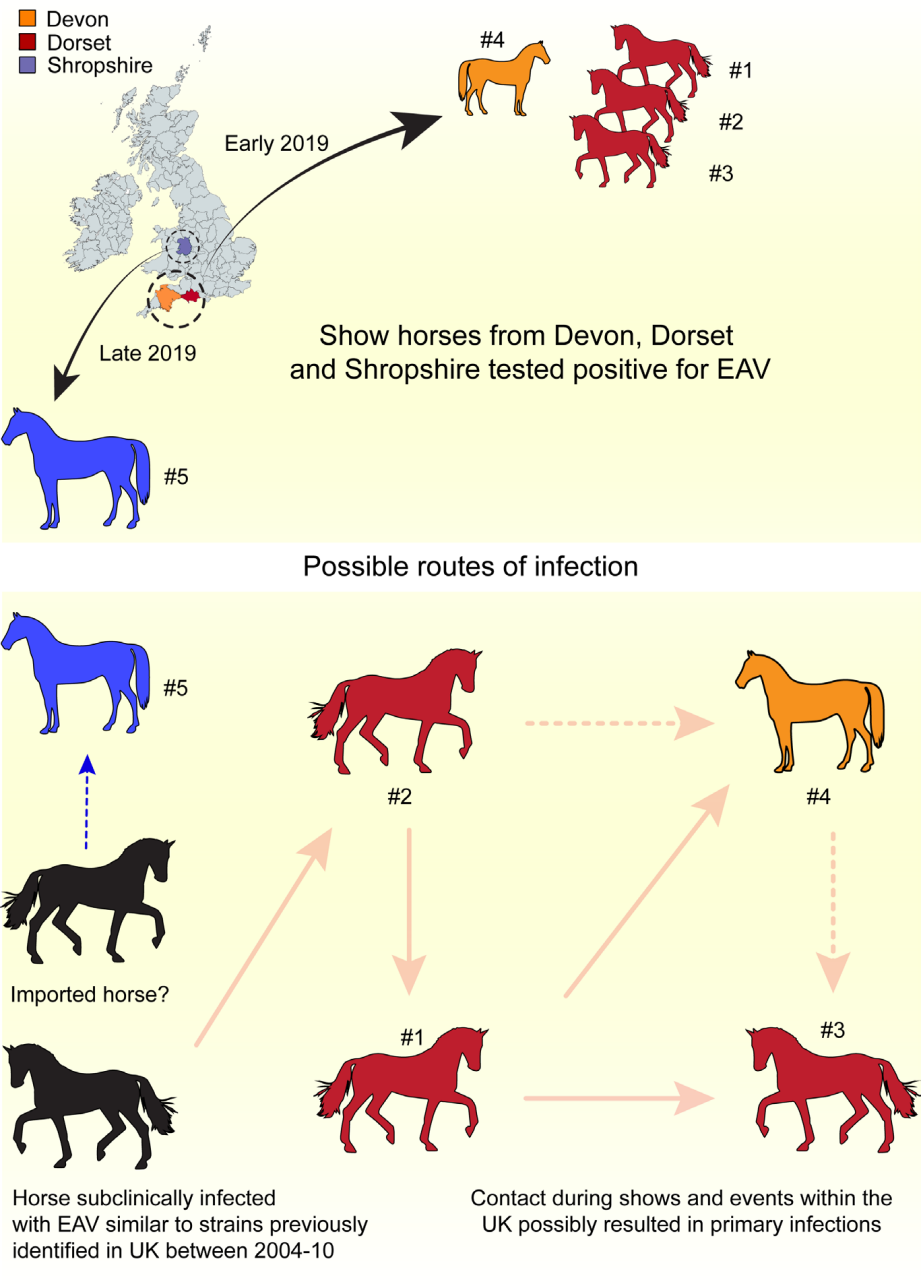
Graphical representation of the EAV outbreak cases in Devon and Dorset. Devon and Dorset dressage stallions are hypothesized to have close contact during transport while horses were moved for shows during 2018 equine gatherings or particularly for breeding purposes. Solid lines – most likely transmission; dotted lines – alternative transmission route.

EAV can be spread via fomites, close contact or sexual transmission [5]. Widespread EVA epizootics are uncommon, and most of the EAV infections occur without clinical manifestation and are confirmed by genome detection and seroconversion [5]. While EAV is present in various EU Member States, imported horses could act as a ‘trojan horse’ due to the absence of statutory testing in many countries. If stallions are not used for breeding, asymptomatic horses may go unnoticed and may spread the disease via equine events of various kinds. The initial sources of infection leading to the EAV outbreaks in the UK in 2019 have not been determined, but the Shropshire case related best to an infection spread from outside the UK. Phylogenetic analysis showed a within-outbreak spread from Horse 2 to the other horses in the Dorset/Devon cluster (illustrated in Fig. 6). Since there is a usual practice of stabling horses together at show events, close contact can occur, thereby enabling the origin of an outbreak and spread of infection. Analyses of ORF5 sequences show that the 2019 Dorset/Devon cases could be linked to previous (2004–2011) EAV outbreaks in the UK. Thus, the presence of carrier stallions who were subclinically infected between 2004 and 2011 and transmitted the virus to other susceptible horses in 2019 cannot be negated. Equally, further circulation and re-introduction of the virus from other countries is possible. Genotyping of the CXCL16 gene in horses affected by the 2019 outbreak revealed that the majority carried the ‘heterozygous susceptible’ genotype, which is significantly associated with an increased risk of becoming long-term carriers of EAV transmission [6,44]. Detection of EAV during routine pre-breeding testing is formally classified as an outbreak, while not necessarily leading to active disease, and can provide important epidemiological insight into the presence of infected carrier stallions spreading virus within the local horse population. Such carriers play a critical role in virus maintenance and dissemination not only during breeding activities. This highlights ongoing virus circulation and underlines the importance of continuous surveillance to prevent potential transmission events that could result in future outbreaks.

In conclusion, the 2019 EAV outbreak reinforces the importance of surveillance, rapid response and effective implementation of control measures to mitigate the spread of EAV. It also underpins the importance of a multifaceted approach involving veterinary surgeons, diagnostic laboratories and horse premises in effectively controlling and managing such outbreaks. Screening breeding stallions alone is insufficient to prevent the transmission of EAV. To effectively control the spread of the virus, particularly in subclinical forms, it is essential to extend testing to non-breeding stallions. A robust DIVA test would further assist in differentiating infected horses from vaccinated ones.

## Supplementary material

10.1099/jgv.0.002181 Uncited Supplementary Material 1.

## Abbreviations

CPE: cytopathic effects
EAV: equine arteritis virus
EMEM: Eagle's minimal essential medium
EVA: equine viral arteritis
FEL: fixed effects likelihood
FUBAR: fast unconstrained Bayesian approximation
GP: glycoprotein
MCMC: markov chain monte carlo
MEME: mixed effects model of evolution
ML: maximum likelihood
nAbs: neutralizing antibodies
nsp: non-structural protein
PNI: percentage nucleotide identity
PRRSV: porcine reproductive and respiratory syndrome virus
qRT-PCR: quantitative reverse transcription PCR
RK13: rabbit kidney 13
SLAC: single-likelihood ancestor counting
SNT: serum neutralization test
