# Supplementary material for: Genetic characterization of equine arteritis virus associated with outbreaks in the UK, 2019
Source: J Gen Virol. 2025 Dec 3;106(12):002181. doi: 10.1099/jgv.0.002181 (PMC12674535; doi:10.1099/jgv.0.002181)
Supplement: Uncited Supplementary Material 1. [file jgv-106-02181-s001.pdf]

Supplementary Figures:

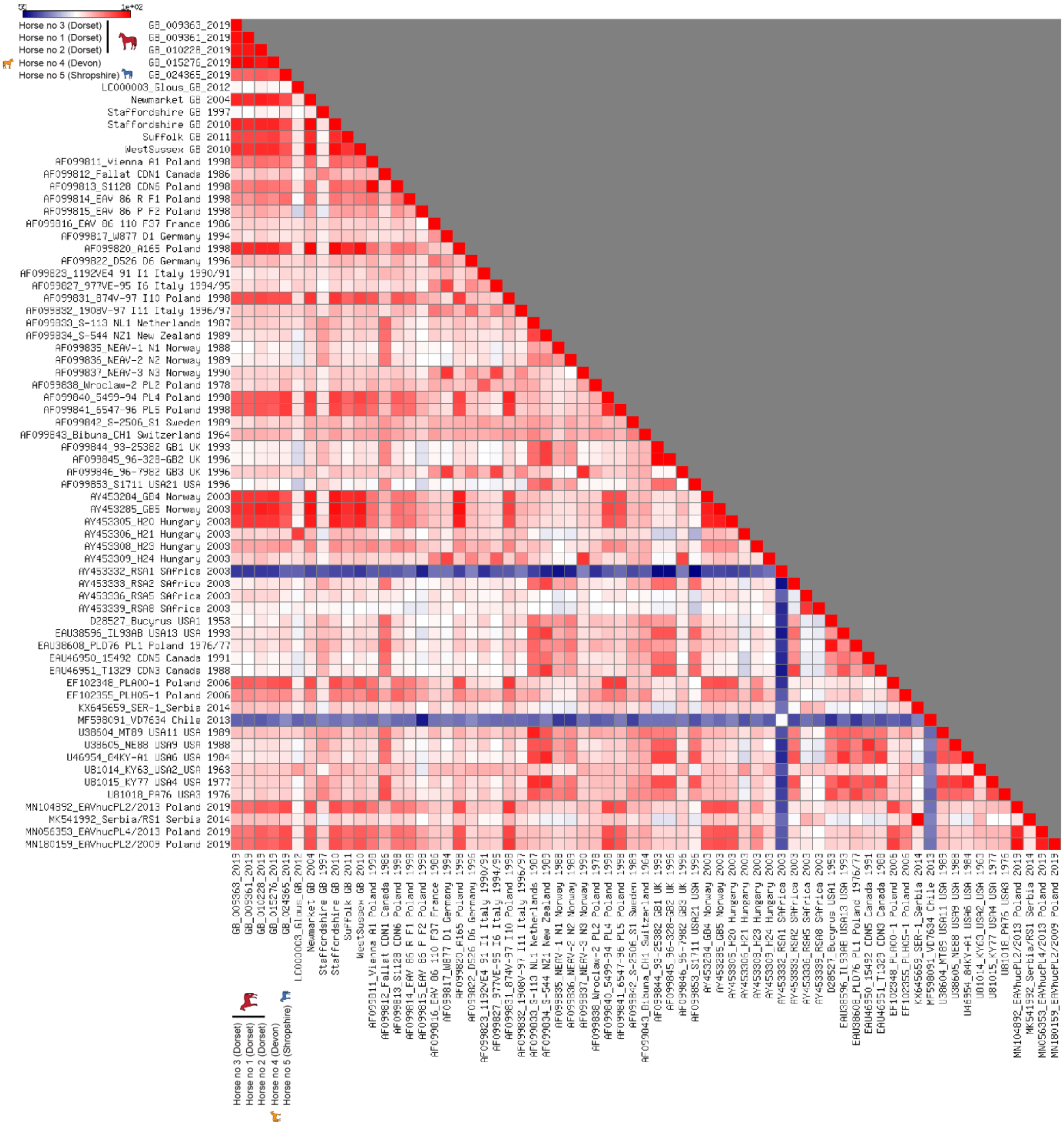

Supplementary Figure S1. Estimates of evolutionary divergence between ORF5 sequences of the equine arteritis strains identified in 2019 in the UK. The number of base substitutions per site from between ORF5 sequences is shown. Pairwise distance(s) were obtained by a bootstrap procedure (1000 replicates), converted to percentage similarity, and are shown below the diagonal. The colouring scale correspond to least similarity or maximum divergence (55%) shown as dark blue, medium similarity or intermediate divergence (77%) shown as white and little to no divergence or maximum similarity (100%) shown as dark red. Analyses were conducted using the Maximum Composite Likelihood model. The analysis involved 67 nucleotide sequences. There were a total of 434 positions in the final dataset. Evolutionary analyses were conducted in MEGA X. The reference sequences include the NCBI accession number followed by the strain name.

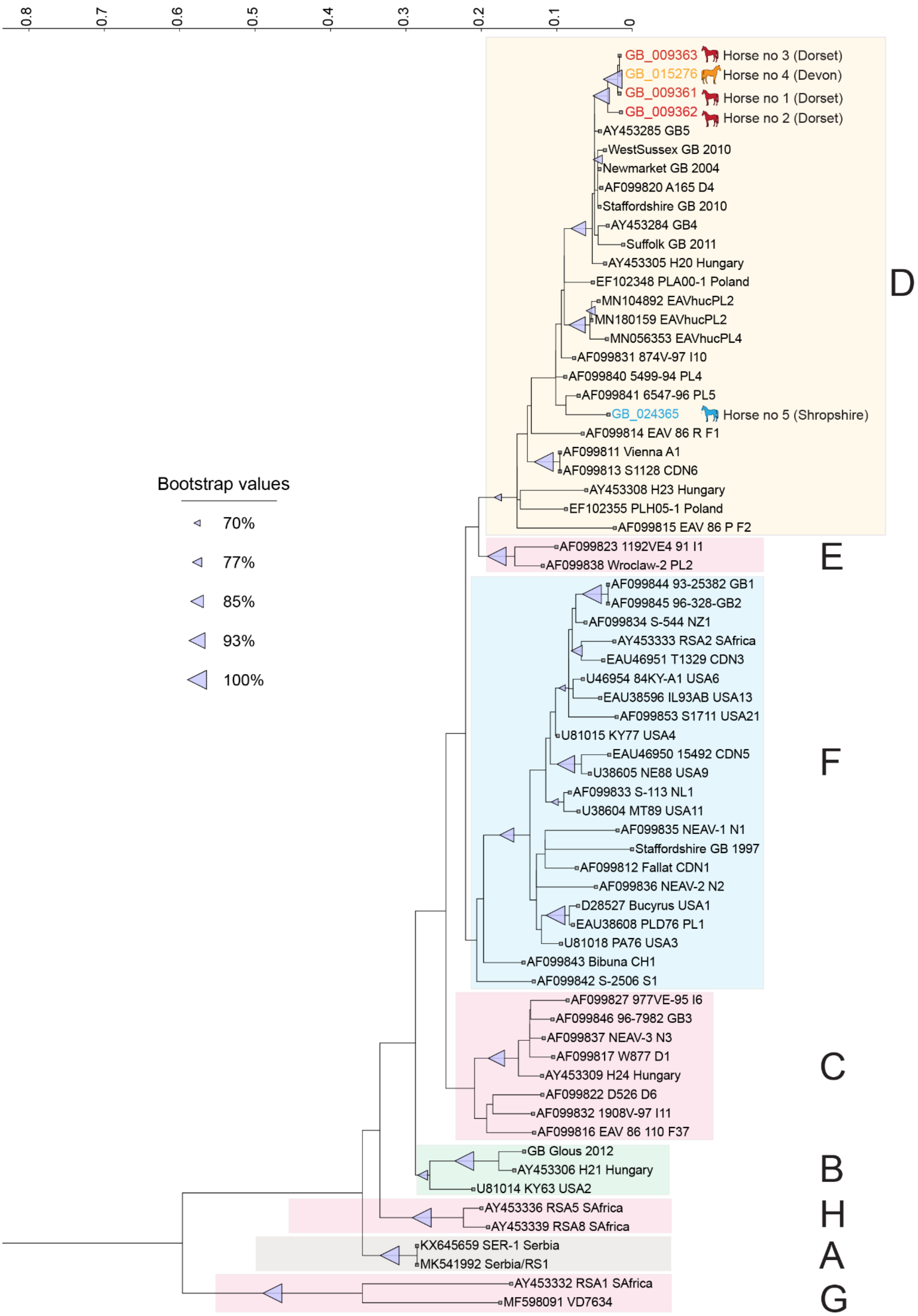
0.8
0.7
0.6
0.5
0.4
0.3
0.2
0.1
0
GB_009363 Horse no 3 (Dorset)
GB_015276 Horse no 4 (Devon)
GB_009361 Horse no 1 (Dorset)
GB_009362 Horse no 2 (Dorset)
AY453285 GB5
WestSussex GB 2010
Newmarket GB 2004
AF099820 A165 D4
Staffordshire GB 2010
AY453284 GB4
Suffolk GB 2011
AY453305 H20 Hungary
EF102348 PLA00-1 Poland
MN104892 EAVhucPL2
MN180159 EAVhucPL2
MN056353 EAVhucPL4
AF099831 874V-97 I10
AF099840 5499-94 PL4
AF099841 6547-96 PL5
GB_024365 Horse no 5 (Shropshire)
AF099814 EAV 86 R F1
AF099811 Vienna A1
AF099813 S1128 CDN6
AY453308 H23 Hungary
EF102355 PLH05-1 Poland
AF099815 EAV 86 P F2
D
AF099823 1192VE4 91 I1
AF099838 Wroclaw-2 PL2
E
AF099844 93-25382 GB1
AF099845 96-328-GB2
AF099834 S-544 NZ1
AY453333 RSA2 SAfrica
EAU46951 T1329 CDN3
U46954 84KY-A1 USA6
EAU38596 IL93AB USA13
AF099853 S1711 USA21
U81015 KY77 USA4
EAU46950 15492 CDN5
U38605 NE88 USA9
AF099833 S-113 NL1
U38604 MT89 USA11
AF099835 NEAV-1 N1
Staffordshire GB 1997
AF099812 Fallat CDN1
AF099836 NEAV-2 N2
D28527 Bucyrus USA1
EAU38608 PLD76 PL1
U81018 PA76 USA3
AF099843 Bibuna CH1
AF099842 S-2506 S1
F
AF099827 977VE-95 I6
AF099846 96-7982 GB3
AF099837 NEAV-3 N3
AF099817 W877 D1
AY453309 H24 Hungary
AF099822 D526 D6
AF099832 1908V-97 I11
AF099816 EAV 86 110 F37
C
GB Glous 2012
AY453306 H21 Hungary
U81014 KY63 USA2
B
AY453336 RSA5 SAfrica
AY453339 RSA8 SAfrica
H
KX645659 SER-1 Serbia
MK541992 Serbia/RS1
A
AY453332 RSA1 SAfrica
MF598091 VD7634
G
Bootstrap values
70%
77%
85%
93%
100%

Supplementary Figure S2 – Phylogenetic analysis of the ORF5 sequences of the EAV strains identified in 2019 in the UK using Maximum Likelihood method. The evolutionary history was inferred using the Maximum Likelihood method and General Time Reversible (GTR+G+I) model. The numbers represent the percentage of times particular branch patterns were predicted after 1000 bootstrap replicates. This analysis involved 67 nucleotide sequences. All positions containing gaps and missing data were eliminated (complete deletion option). Evolutionary analyses were conducted in MEGA X. The reference sequences include the NCBI accession number followed by the strain name.

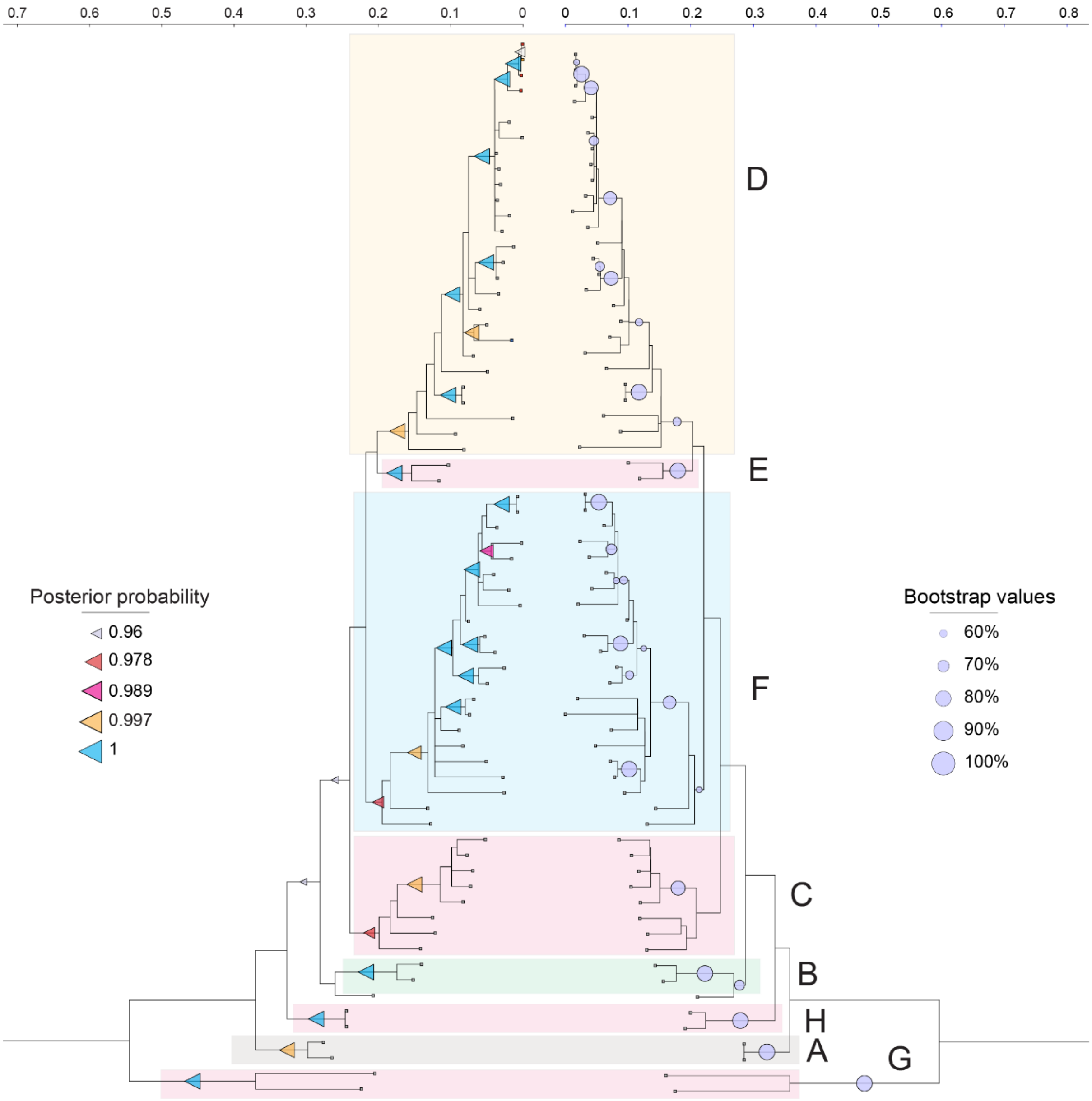

Supplementary Figure S3 – Head-on comparison of different phylogroups identified using Markov chain Monte Carlo (MCMC) and Maximum Likelihood (ML) method

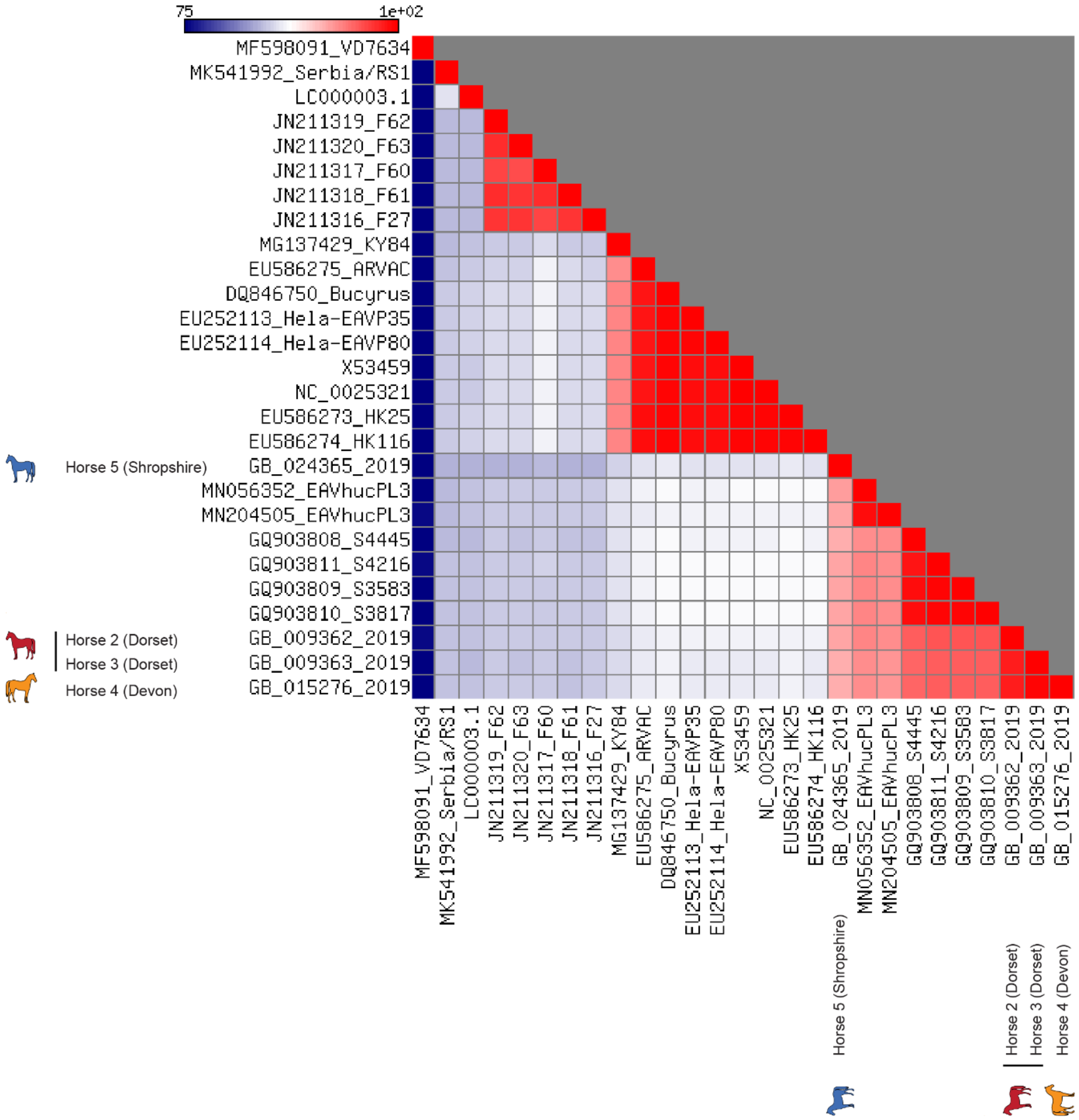

Supplementary Figure S4. Estimates of evolutionary divergence between whole genome sequences of the equine arteritis strains identified in 2019 in the UK. The number of base substitutions per site from between whole genome sequences is shown. Pairwise distance(s) were obtained by a bootstrap procedure (1000 replicates), converted to percentage similarity, and are shown below and above the diagonal. The colouring scale corresponds to least similarity or maximum divergence (75%) shown as dark blue, medium similarity or intermediate divergence (87.5%) shown as white and little to no divergence or maximum similarity (100%) shown as dark red. Analyses were conducted using the Maximum Composite Likelihood model. This analysis involved 28 full genome nucleotide sequences. There were a total of 12454 positions in the final dataset. Evolutionary analyses were conducted in MEGA X. The reference sequences include the NCBI accession number followed by the strain name.

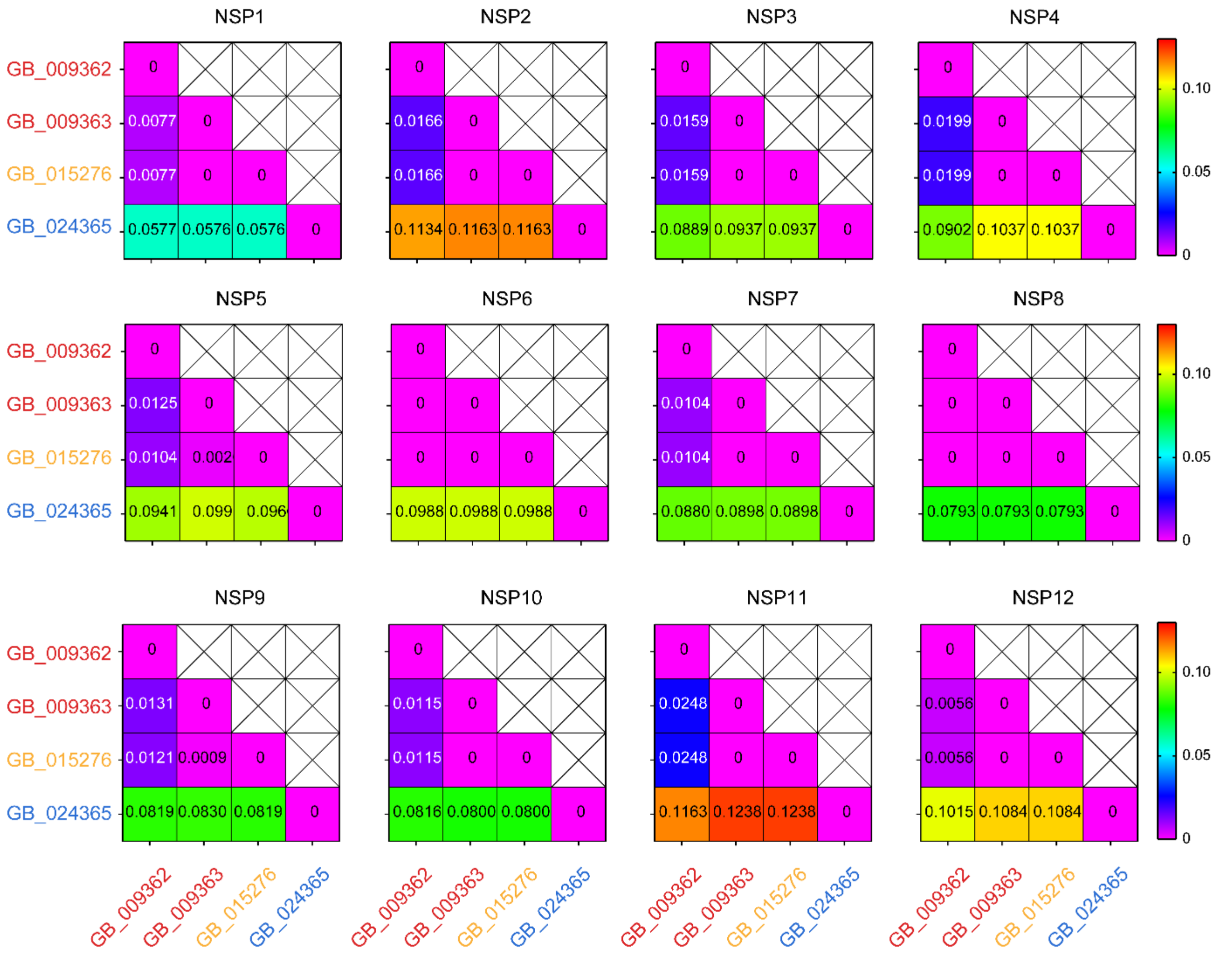

Supplementary Figure S5. Estimates of evolutionary divergence between different non structural protein (nsp) sequences of the equine arteritis strains identified in 2019 in the UK.

The number of nucleotide substitutions per site from different nsp sequences is shown. Pairwise distance(s) were obtained by a bootstrap procedure (1000 replicates), in MEGA X, and the values are shown below the diagonal in each cell. The pairwise distance was plotted as a heatmap using GraphPad Prism 8. The accession numbers of the strains used in the analysis is shown on the left side and the bottom of the heat map. E: envelope protein; GP: glycoprotein; M: membrane protein; N: nucleocapsid protein; nsp: non-structural protein; ORF: Open Reading Frame.

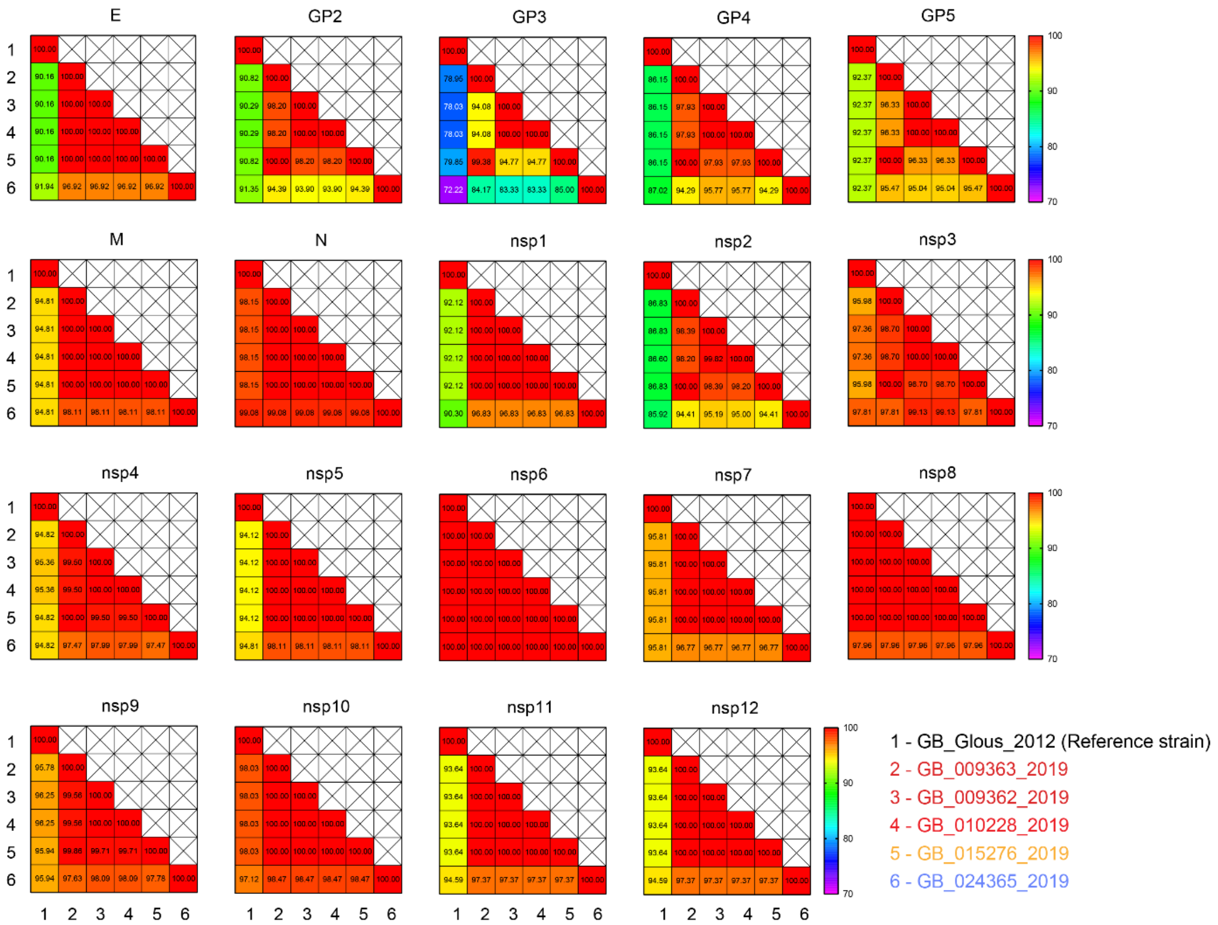

Supplementary Figure S6. Estimates of evolutionary divergence between different protein sequences of the equine arteritis strains identified in 2019 in the UK.

The number of amino acid substitutions per site from between different gene sequences is shown. Pairwise distance(s) were obtained by a bootstrap procedure (1000 replicates), in MEGA X and converted to percentage similarity, and the values are shown below the diagonal in each cell. The percentage similarity was plotted as a heatmap using GraphPad Prism 8. The numbers 1-6 on the left side and the bottom of the heat map represent the isolate ID of the strains used in the analysis. The full genome sequence from an EAV isolate identified previously in 2012 in Gloucestershire, UK (GB_Glous_2012) was taken as a reference for comparison. E: envelope protein; GP: glycoprotein; M: membrane protein; N: nucleocapsid protein; nsp: non-structural protein.

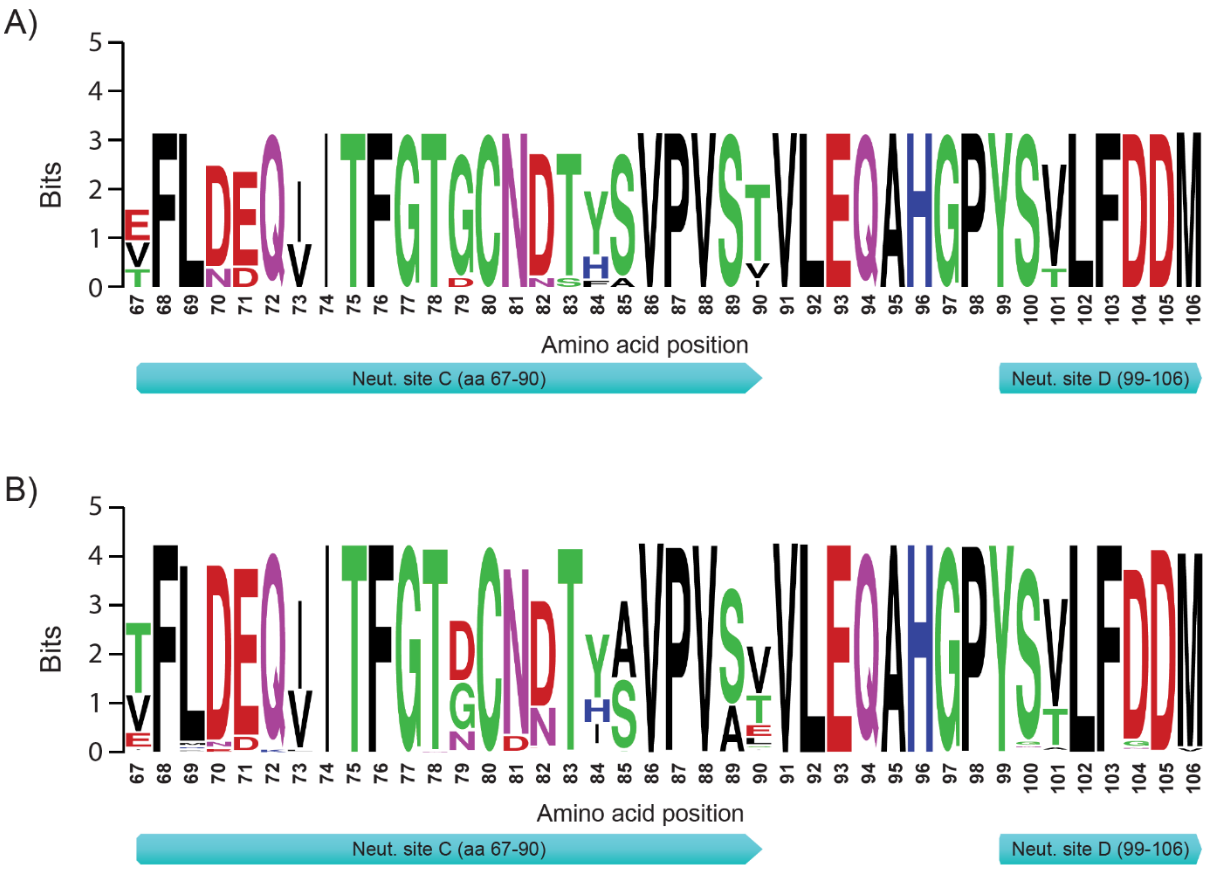

Supplementary Figure S7. Amino acid analysis of neutralisation sites C and D present in GP5 of EAV 2019 virus strains is shown as a sequencing logo. A) shows amino acid differences between the 2019 disease outbreak strains and previous EAVs identified in the UK. B) shows amino acid differences between the EAVs identified worldwide. The analysis for 'A' was carried out using five EAV 2019 outbreak strains and seven EAVs previously reported in the UK (compare Fig 1), while the analysis for 'B' involved 597 GP5 sequences retrieved from the Genbank (excluding the sequences containing ambiguous nucleotides). Each logo consists of a letter or a stack of letters representing an amino acid at the corresponding amino acid position in neutralisation site C (aa 67-90) or neutralisation site D (aa 99-106). The overall height of the stack (shown as bits) indicates the sequence conservation at that position. The height of the symbols within each stack represents the relative frequency of each amino acid at that position. Amino acids are coloured according to their chemical properties: polar amino acids (G, S, T, Y, C, Q, N) are green, basic (K, R, H) are blue, acidic (D, E) are red and hydrophobic (A, V, L, I, P, W, F, M) amino acids are shown as black.

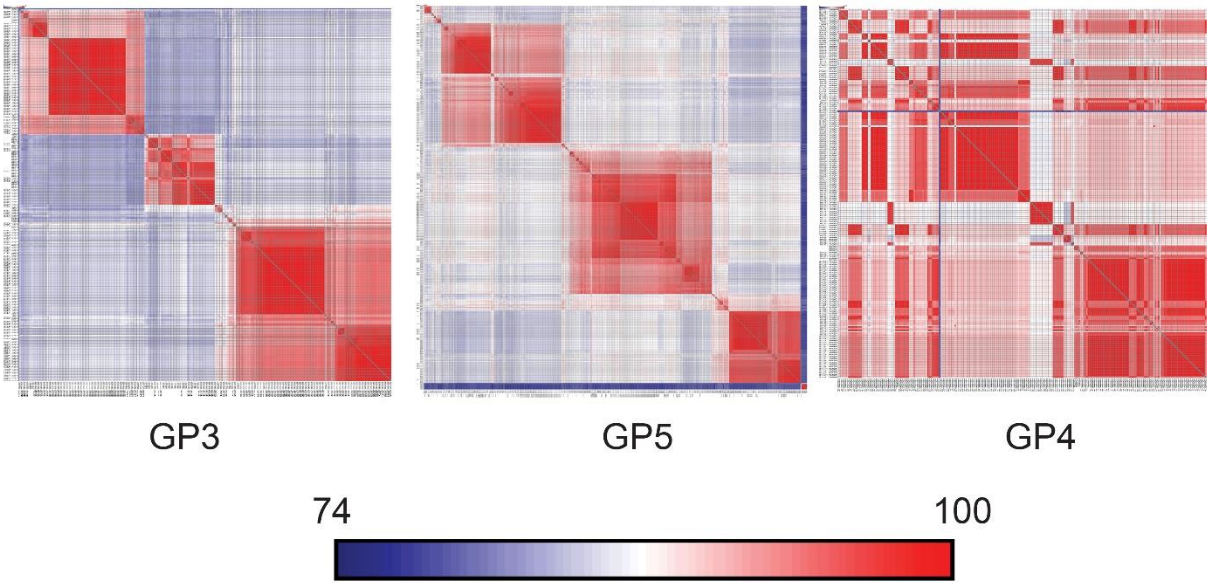

Supplementary Figure S8: **Estimates of evolutionary divergence between different ORF3 (GP3), ORF5 (GP5) and ORF4 (GP4) sequences of the equine arteritis strains identified globally.** The number of base substitutions per site between ORF3(GP3), ORF5 (GP5) and ORF4 (GP4) sequences is shown. Pairwise distance(s) were obtained by a bootstrap procedure (1000 replicates), in MEGA X and converted to percentage similarity, and the values are shown below the diagonal in each cell. The colouring scale corresponds to least similarity or maximum divergence (74%) shown as dark blue, medium similarity or intermediate divergence (87%) shown as white and highest similarity or least divergence (100%) shown as dark red. The analysis involved 296, 623 and 243 nucleotide sequences for GP3, GP5 and GP4 respectively. For GP5 sequences, the alignment containing missing data due to partial GP5 sequences was deleted. There were a total of 504, 520 and 466 positions in the final dataset for GP3, GP5 and GP4, respectively. All the reference sequences include the NCBI accession number. The heat maps are arranged starting with the maximum divergence.

Supplementary Table S1: Selection pressure analysis of ORF5 (GP5 protein) of EAV using SLAC, FEL, MEME and FUBAR methods showing codons undergoing positive or diversifying selection

Codon	SLAC		FEL		MEME		FUBAR	
	dN-dS	P-value	β	P-value	β+	P-value	Prob (α<β)	Bayes factor (α<β)
3	2.895091	0.000052	1.531	0	11.055	0	1	36872.022
14	2.080962	0.000202	1.142	0.0001	8.346	0	1	10333.718
36	1.861283	0.000825	1.135	0.0008	8.213	0.002	0.999	3333.325
61	5.289501	0.000528	4.483	0.0002	55.757	0	0.999	3061.267
82	3.154706	0.004701	2.832	0.006	20.605	0.01	1	6798.895
101	2.656267	0.000098	1.533	0.0001	106.599	0	0.999	2528.895
104	1.187853	0.041794	0.677	0.0072	46.65	0	0.991	265.496
119	1.270697	0.051139	1.004	0.017	28.798	0.009	0.916	27.82
236	1.37484	0.00396	0.751	0.0013	36.674	0.001	0.988	212.836

Supplementary Table S2: Selection pressure analysis of ORF3 (GP3 protein) of EAV using SLAC, FEL, MEME and FUBAR methods showing codons undergoing positive or diversifying selection

Codon	SLAC		FEL		MEME		FUBAR	
	dN-dS	P-value	β	P-value	β+	P-value	Prob (α<β)	Bayes factor (α<β)
3	2.205499	0.016602	1.775	0.0264	12.285	0.036	0.991	153.177
4	1.399392	0.026012	0.799	0.0043	4.074	0.008	0.989	124.53
5	1.081159	0.096391	0.73	0.0141	3.773	0.022	0.975	54.862
6	2.095366	0.011689	1.294	0.009	6.646	0.015	0.998	579.652
9	1.554184	0.017419	1.094	0.0073	5.603	0.012	0.989	129.538
10	1.560325	0.01714	0.999	0.0017	5.121	0.003	0.993	187.086
16	1.641061	0.018222	1.191	0.001	6.146	0.002	0.998	640.069
18	1.533698	0.038357	1.235	0.0119	6.581	0.019	0.992	175.265
19	3.60463	0.000181	2.931	0	100.933	0	1	244541.779
20	1.281519	0.057426	1.035	0.0035	5.317	0.006	0.993	184.259
21	1.057124	0.071787	0.648	0.0126	3.34	0.02	0.976	55.417
22	3.316642	0.000357	2.68	0.0001	13.702	0	1	2925.72
23	1.337139	0.066525	1.156	0.0171	5.986	0.026	0.983	81.223
24	1.66774	0.029686	1.008	0.0209	17.225	0.013	0.985	91.383
25	1.999164	0.006083	1.565	0.0017	17.142	0.002	0.999	1795.085
27	3.72116	0.00023	2.376	0	38.975	0	1	56132.081
30	1.680932	0.014018	0.894	0.0146	107.073	0	0.98	66.32
39	2.401847	0.005172	1.455	0.0005	7.51	0.001	1	16518.666
120	4.407235	0.003332	3.507	0.0151	71.304	0.001	0.997	507.742
123	3.134297	0.060927	3.544	0.0098	18.272	0.016	0.988	116.501
